# 

**Published:** 1906-05-05

**Authors:** 


CONTENTS.

**Euthanasia** - - - - 79
**Annotations** - - - - 80
**Hospital Clinics—**
The Rôle of Alcohol in the Treatment of Certain Diseases - - 81
Discussion on Syphilis - - - - 83
Endemic Goitre - - - - 84
Obstacles to Hygienic Achievement - - - 85
Amaurotic Family History - - - - 86
**Progress in Medicine and Surgery—**
Rheumatism - - - - 86
X-Rays and Blood - - - - 87
**Professor Sims Woodhead at the Hospital Saturday Fund** - - - 89
**The Book World of Medicine and Science** - - 90
**New Appliances and Things Medical** - - - 91
**Medical Appointments and Vacancies** - - 91
**Hospital Administration—**
The Fifteenth International Congress of Medicine - - 92
Isolation Hospital for the Ampthill District of Bedfordshire - 93
The Shortcomings of Plenum Ventilation - - 94
Hospital Saturday Fund - - - 95
Hospital Meetings - - - - 95
**Notes and News** - - - - 96

*NURSING SECTION.*

**Notes on News from the Nursing World—** Honours for Nigerian Nurses—Outdoor Uniform—A Notable Jubilee—On Duty Fourteen Hours—Alleged Opening for English Nurses in Belgium—Four Years' Training at a Poor-law Infirmary—A Catastrophe at Derbyshire Infirmary—Extraordinary Action at Meath Infirmary—The Salary Question at Edmonton—District and Private Nursing—The Pension Fund and Kentish Nurses—Imperial Military Nursing Service—Guy's Hospital Musical Society—Art and District Nursing—Memorial Home in Glasgow—An Excellent Example at Lowestoft—Short Items - - - 71-74
**The Nursing Outlook** - - - 75
**The Care and Nursing of the Insane** - - - 76
**The Nurses' Clinic** - - - 77
**Incident in the Life of an Irish Nurse** - - 79
**Queen Victoria's Jubilee Institute for Nurses** - 78
**Central Midwives Board** - - - 79
**Everybody's Opinion** - - - 80
**Presentations** - - - 81
**Novelties for Nurses** - - - 81
**Appointments** - - - 81
**A Book and Its Story** - - - 82
**Notes and Queries** - - - 83
**For Reading to the Sick** - - - 84

